# Draft Genome Sequence of Paenibacillus Strain P1XP2, a Polysaccharide-Degrading, Thermophilic, Facultative Anaerobic Bacterium Isolated from a Commercial Bioreactor Degrading Food Waste

**DOI:** 10.1128/genomeA.01484-14

**Published:** 2015-01-29

**Authors:** Joseph Adelskov, Bharat K. C. Patel

**Affiliations:** Microbial Gene Research and Resources Facility, School of Natural Sciences, Griffith University, Brisbane, Queensland, Australia

## Abstract

The analysis of the ~5.8-Mb draft genome sequence of a moderately thermophilic, heterotrophic, facultative anaerobic bacterium, Paenibacillus strain P1XP2, identified genes for enzymes with the potential for degrading complex food wastes, a property consistent with the ecological habitat of the isolate.

## INTRODUCTION

Paenibacillus strain P1XP2 (KCTC 33553; JCM 30052) is a heterotrophic, moderately thermophilic (optimal growth temperature of ~45°C and optimal growth pH between 7 and 8), facultative anaerobic, and fermentative bacterium isolated on 0.1% tryptic soy broth (TSB) from a sample collected from Baku Baku, a commercial bioreactor degrading food waste (1). The sample was collected on day 16 following the commissioning of the reactor, when the *in situ* temperature of the bioreactor was 42°C. Strain P1XP2 was one of 5 strains isolated from the bioreactor that shared 99% of its 16S rRNA gene sequence with Paenibacillus cookii, whose genome is yet to be sequenced. Strain P1XP2 was cultured in 0.1% TSB under optimal growth conditions (pH 7.0 and 45°C) to mid-late log growth phase, the cells were centrifuged, and the DNA from the cell pellet was purified using a modification of Marmur’s method (2). The genomic DNA of strain P1XP2 consisting of 2.1 million (2,093,889) reads totaling 408 Mbp (408,551,646 bp) was generated using an Ion Torrent PGM sequencer and a 318 chip at the Australian Genome Research Facility (AGRF) core facility. Filtered high-quality data assembled using GS De Novo Assembler version 2.9 generated a genome of ~5.8 Mbp (70× coverage) consisting of 186 contigs with an average G+C content of 53.5 mol%. The contigs were analyzed using the online annotation server RAST (3), and automatic gene annotation was carried out using Prokka version 1.10 (4). Phylogenetic analysis for marker genes was performed using Phylosift version 1.0.0_02 (5), and average nucleotide analysis was performed using JSpecies (6).

RAST analysis indicated that the genome sequence comprised 6,608 putative protein-encoding genes, 81 tRNA genes, and 5 rRNA genes (5S rRNA, 16S rRNA, and 23S rRNA). A large number of genes involved in carbohydrate degradation (639), including genes for chitinase, α-amylase, pullulanase, cyclodextrinase, glucosidase, galactosidase, glucuroxylanase, and arabinogalactan endo-1,4-beta-galactosidase, together with a slightly smaller number of genes (339) involved in protein and amino acid degradation and synthesis, were identified in the genome following RAST analysis. The presence of xylanase and amylase in strain P1XP2 was supported by activity screening using X-xyl and starch agar plate assays (1). The presence of chitinase genes has not been reported in any of the 53 Paenibacillus genomes sequenced to date, but their presence has been reported in a number of Paenibacillus isolates (7). Phylogenetic analysis indicated that 97% of the phylogenetic marker genes were related to members of the genus Paenibacillus, family *Paenibacillaceae*, phylum *Firmicutes*, and 10% of these were related to the genome sequence of Paenibacillus sochii. ANIb analysis suggested that strain P1XP2 was distantly related to the 53 genome sequences of Paenibacillus, with an average nucleotide identity of 74%, suggesting that it is a novel isolate.

The draft whole-genome sequence of strain P1XP2, together with isolate Bacillus subtilis strain D7XPN1, another potent enzyme producer from the same ecosystem and whose genome has been sequenced (1), will assist in understanding the role of this particular bacterium in the food-degradation process in the Baku Baku commercial bioreactor.

### Nucleotide sequence accession numbers.

The Paenibacillus sp. strain P1XP2 whole-genome shotgun project has the project accession number JRNV00000000. This version of the project (01) has the accession number JRNV01000000 and consists of sequences JRNV01000001 through JRNV01000186. The version described in this paper is version JHCA00000000.1.
